# Title: Discrepancies in Anxiety Ratings Between Children with ADHD and Their Parents: The Impact of Sensory-Motor Dysfunction

**DOI:** 10.1192/j.eurpsy.2025.1140

**Published:** 2025-08-26

**Authors:** T. Gur-Hartman, R. Tarrasch, A. Zerem, R. Sokol-Novinsky, Z. Elyoseph, T. Lerman-Sagie, M. Mintz

**Affiliations:** 1 School of Psychological Sciences, Tel Aviv University, Tel Aviv; 2 Pediatric Neurology Unit, Wolfson Medical Center, Holon; 3 Sagol School of Neuroscience; 4 School of Education; 5 Faculty of Medicine, Tel Aviv University; 6 Pediatric Neurology Institute, Dana-Dwek Children’s Hospital, Tel Aviv; 7 Academic College of Emek Yezreel, Afula; 8 Faculty of Medicine, Tel Aviv University; 9 Pediatric Neurology Unit, Wolfson Medical Center, Tel Aviv, Israel

## Abstract

**Introduction:**

Children with Attention Deficit Hyperactivity Disorder (ADHD) often experience comorbid anxiety. While their anxiety is typically assessed through both children’s and parents’ reports, discrepancies between these reports are common and poorly understood. Children with ADHD also present sensory-motor dysfunctions, of which explicit expressions may mask children’s emotional state.

**Objectives:**

Here, we test whether parents of children with sensory-motor dysfunctions may underestimate their children’s anxiety levels.

**Methods:**

Participants were 85 children: ADHD-only (n=28), ADHD+DCD (n=38), and typically developing (TD; n=19). Hyperactivity/Impulsivity was assessed by DSM-V. Anxiety was assessed by children and parents using the Screen for Child Anxiety-Related Disorders questionnaire (SCARED-C/P). Sensory-motor functions were evaluated using the Movement Assessment Battery for Children (MABC-2), balance subtest of the Bruininks Osteresky Test of Motor Proficiency (BOT-2), and vestibular tests of Dynamic Visual Acuity (DVAt) and standing posturography.

**Results:**

Anxiety ratings were higher by children with ADHD than TD children (t-tests, 0.001<p<0.05). Anxiety ratings were higher by children than their parents (t-tests, 0.001<p<0.05), and the correlation between child and parent anxiety ratings was lower in ADHD dyads (0.22<r<0.33, p<0.05) compared to TD dyads (0.60<r<0.70, p<0.01). Panic anxiety ratings by children correlated positively with vestibular dysfunction (r=0.22, p<0.05), indicating that anxiety ratings increase with vestibular dysfunction. Anxiety ratings by parents correlated negatively with motor dysfunctions on the vestibular, postural stability, balance, and motor function tests (-0.36<r<-0.22, p<0.05), indicating that anxiety ratings decrease with motor deficiency. Finally, the ADHD cohort was assigned to an “agreement” cluster (55%) with comparable child-parent anxiety ratings and to a “disagreement” cluster (45%) with anxiety ratings lower by parents than children. Children in the “disagreement” vs. “agreement” cluster demonstrated higher hyperactivity/impulsivity scores by DSM-V (p<0.05) and lower motor performance by MABC-2 (p<0.05).

**Conclusions:**

Findings suggest that parents of children with ADHD underestimate their child’s anxiety, mainly when children’s sensory-motor symptoms are prominent. We summarize these results under the term “blinding effect,” which implies that parents’ primary concern is the child’s sensory-motor dysfunction, thus neglecting the expressions of anxiety. This “blinding effect” should be studied in the context of additional neurodevelopmental disorders sharing sensory-motor dysfunctions, and clinicians should be aware of the potential child-parent bias in reporting anxiety.

**Disclosure of Interest:**

None Declared

